# Potential utility of PPARγ agonists in targeting chronic myeloid leukemia stem cells

**DOI:** 10.1007/s00277-026-06795-7

**Published:** 2026-02-25

**Authors:** Basma Atef, Shaimaa El-Ashwah, Layla M. Saleh, Hanan Gawish, Mohamed Mabed

**Affiliations:** 1 Hematology Unit, Internal Medicine Department, Faculty of Medicine, Mansoura University, Mansoura, Egypt; 2 Clinical Pathology Department, Faculty of Medicine, Hematology Unit, Mansoura University, Mansoura, Egypt; 3 Diabetes & Endocrinology Unit, Internal Medicine Department, Faculty of Medicine, Mansoura University, Mansoura, Egypt

**Keywords:** Chronic myeloid leukemia, Leukemic stem cells, PPARγ agonists, Pioglitazone, *CITED2* gene, *HIF2α* gene

## Abstract

Tyrosine kinase inhibitors (TKIs) have transformed the treatment of chronic myeloid leukemia (CML), yet persistent leukemia stem cells (LSCs) remain a barrier to cure. PPARγ agonists like pioglitazone have been proposed to enhance eradication of LSCs when used alongside TKIs. This study investigated the impact of adding pioglitazone to imatinib therapy in 26 newly diagnosed chronic-phase CML patients. Patients received imatinib (400 mg) plus pioglitazone (15 mg) daily for six months, with follow-up extending to 60 months. Treatment responses and adverse events were recorded, and expression levels of *CITED2* and *HIF2α* genes were measured before and after therapy, compared to a control group of 52 matched patients treated with imatinib alone. The combination therapy showed improved early cytogenetic and molecular responses, though long-term outcomes were not significantly different. Significant reductions in median *CITED2* (from 276.3 to 2.6; *P* = 0.005) and *HIF2α* (from 2.7 to 1; *P* = 0.026) expression were observed post-treatment. These results suggest that pioglitazone may enhance early molecular response and suppress LSC-associated genes, but further research is needed to confirm its long-term benefit and clarify the role of PPARγ modulation in CML management. **Clinical Trial Number:** NCT04883125.

## Introduction

Chronic myeloid leukemia (CML) is a myeloproliferative disorder that is characterized by the cytogenic hallmark "Philadelphia chromosome (Ph +)". The use of tyrosine kinase inhibitors (TKIs) has revolutionized the treatmentof CML and improved the patients’ survival dramatically [1]**.** Moreover, the obtained deep molecular responses have encouraged starting the treatments free remission (TFR) trials. However, 50% of the patients relapse [2]**.** Researches owed this relapse mainly to the presence of quiescent/therapy resistant leukemic stem cells (LSCs) [3]. They also proved that LSCs are “Not oncogene-addicted” i.e. their survival is not strictly dependent on BCR::ABL1 kinase activity alone but on many other signaling pathways [4]**.** Studies have reported how strong the extra-activated STAT5 can regulate the quiescence/proliferation of LSCs through many target genes for example: Hypoxia inducible factor genes and *CITED2* genes [5, 6]**.**

Peroxisome proliferator-activated receptor gamma (PPARγ) is one of nuclear-hormone receptors that regulate cellular differentiation, proliferation and apoptosis. Combining pioglitazone (PPARγ agonist) with imatinib has increased the anti-leukemic effect in vitro [7–9] and in vivo studies [5, 10]**.** This was explained by its ability to down regulate the STAT5 target genes and subsequently impact the LSC cell cycle and increase their sensitivity to therapy [7, 8]**.**

## Aim of the study

The objective of this study was to assess the therapeutic impact of combining glitazones with tyrosine kinase inhibitors (TKIs) in the treatment of newly diagnosed patients with chronic phase chronic myeloid leukemia (CML-CP). The primary endpoint was to evaluate the safety profile of the combination regimen. Secondary endpoints included assessment of short-term (at 3, 6, and 12 months) and long-term treatment responses and efficacy, based on molecular response milestones. The tertiary endpoint involved evaluating changes in the expression levels of the *CITED2* and *HIF2α *genes before treatment and after 6 months of therapy.

## Study design and methodology

### Study design

This was a prospective, non-randomized interventional study conducted at the Oncology Center Mansoura University (OCMU), Egypt, from January 2017 to January 2022. Ethical approval was obtained from the Faculty of Medicine Ethical Committee (MD/17.12.30), and the study was registered as a clinical trial (NCT04883125).

### Study population

The study included 26 newly diagnosed patients with chronic phase Philadelphia chromosome–positive chronic myeloid leukemia (CML), with the sample size calculated to detect a 25% absolute increase in major molecular response (MMR) at 12 months (from 25 to 50%), assuming a significance level (α) of 0.05 and a statistical power of 80% [11]**.** Eligible participants were adults aged 18 to 60 years of both sexes, diagnosed with de novo chronic phase CML, and who provided written informed consent in accordance with the Declaration of Helsinki. Exclusion criteria included advanced disease stages (accelerated or blast phase), atypical CML, and any significant organ dysfunction.

### Diagnosis and treatment

CML diagnosis was confirmed via real-time quantitative polymerase chain reaction (RT-qPCR) for the BCR::ABL gene from peripheral blood mononuclear cells (PBMNCs) (standardized to the International Scale (IS), using ABL1 as the control gene). Bone marrow examination and flow cytometry were performed to evaluate cellularity and rule out advanced stages. Patients received a combination of pioglitazone 15 mg and imatinib 400 mg**,** both once daily, for 6 months, with close clinical monitoring. All clinical data, adverse effects, and treatment responses were recorded in OCMU’s electronic medical records. BCR::ABL transcript levels were monitored by RT-qPCR at 3, 6, 12, 24, 36, 48, and 60 months to evaluate treatment response. Molecular responses were categorized according to log reductions on the International Scale (IS) as follows: Early Molecular Response (EMR) was defined as BCR::ABL IS ≤ 10% at 3 months; Complete Cytogenetic Response (CCyR) corresponded to the absence of Philadelphia chromosome–positive metaphases in ≥ 20 bone marrow cells, approximated by BCR::ABL IS < 1%; Major Molecular Response (MMR) was defined as BCR::ABL IS ≤ 0.1%; Deep Molecular Response (DMR) as BCR::ABL IS ≤ 0.01%; and Complete Molecular Response (CMR) as undetectable BCR::ABL transcripts on the IS. At 6 months responders were defined as patients achieving favourable (BCR::ABL1 ≤ 1%) or warning (BCR::ABL1 > 1–10% IS) responses while non-responders were those who failed to achieve this milestone (BCR::ABL1 > 10%) according to the European Leukemia Net (ELN) recommendations [12]**.**

### Outcomes and evaluation

#### Safety

Treatment safety was defined by the absence of grade 3–4 hematologic or non-hematologic toxicities at any time [13]**.**

#### Efficacy

Efficacy was assessed via the decline in BCR::ABL1⁺transcripts over time and achievement of molecular response milestones**.** Any progression to accelerated or blast phase during follow-up was documented. A **historical control group** was selected for comparing of clinical and molecular outcomes., consisting of 52 age and gender matched patients with chronic-phase Ph⁺ CML who had been treated with imatinib 400 mg daily at OCMU between 2012 and 2016, prior to the initiation of the prospective cohort. Only patients with complete demographic, diagnostic, clinical, and response data on OCMU electronic medical records were included.

#### Gene expression analysis

The relative Expression levels of CITED2 and HIF2α were evaluated before treatment and at 6 months and were compared with levels in a third group of 12 age- and gender-matched healthy blood donors. For analysis of relative gene expression (CITED2, HIF2α, and BCR::ABL1), peripheral blood samples were collected and RNA was extracted from PBMNCs. cDNA was synthesized, and the resultant aliquot was used for gene expression analysis by RT-qPCR (on the StepOne™ Real-Time PCR System). Specific primers for HIF2α and CITED2 were obtained from Applied Biosystems (TaqMan® Gene Expression Assays). Relative gene expression was calculated using the 2^ − ΔΔCt method with GAPDH was used as control gene*.*

#### Statistical analysis

Statistical analysis was performed using SPSS Version 25.0. Normality was tested using the Kolmogorov–Smirnov test (p > 0.05 considered normal). Normally distributed variables were compared using the Student’s t-test, while non-parametric variables were analysed using the Mann–Whitney U test. Chi-square or Fisher’s exact test was used for categorical variables, as appropriate.

## Results

### Demographic data of both groups

The study group comprised 15 males (57.7%) and 11 females (42.3%), with a mean age of 43.2 ± 12.7 years. The historical control group included 27 males (51.9%) and 25 females (48.1%), with a mean age of 44.5 ± 13.3 years. No statistically significant differences were observed between the two groups in terms of demographic characteristics, clinical parameters, laboratory findings, or the various risk scoring systems **[**Table 1**]**.

**Table 1:** Demographic, clinical data and risk scoring of patients among groups

Demographic data	Historical No = 52	Study No = 26	*P*
**Age in years** (Mean ± SD)	44.5 ± 12.7	43.2 ± 13.3	0.700
**Males** **Females**	27 (51.9%) 25 (48.1%)	15 (57.7%) 11 (42.3%)	0.630
**Clinical presentation:** Accidental Leucostasis Manifestations of Anemia Constitutional symptoms Organomegaly	18 (34.6%) 1 (1.9%) 7 (13.5%) 16 (30.8%) 15 (28.8%)	7 (26.9%) 0 (0%) 2 (7.7%) 13 (50%) 11 (42.3%)	0.493 1 0.710 0.098 0.234
**CBC (Median/range):** TLC (X10^9^/L) HB (g/dL) Platelets (X10^9^/L)	124 (10–601) 11 (6.5–15) 251 (23–770)	137.5 (35–249) 10 (7–15) 303.5 (99–829)	0.528 0.228 0.173
**DLC (Median/range):** PMNLs(%) Myelo (%) Meta (%) Basophils Eosino (%) Mono (%) Blast (%)	49.5 (26–74) 14.5 (3–35) 16.5 (1–35) 2 (0–17) 2 (0–10) 1 (0–6) 1 (0–9)	56.5 (0.75–85) 12.5 (3–31) 19 (5–29) 2 (0.1–9) 2 (0.8–7) 1.8 (0–9.7) 1 (0–9)	0.030 0.230 0.620 0.910 0.548 0.393 0.884
**BM Cellularity (Median/range):** Hypercellular/Normocellular Hyocellular	24/4 0	18/4 1	0.416
Bone Marrow Blasts %	4 (1–5)	3 (1–5)	0.114
**Sokal scoring N (%):** Low Intermediate High	28 (53.8%) 20 (38.5%) 4 (7.7%)	13 (50%) 11 (42.3%) 2 (7.7%)	0.933
**Eutos scoring N (%)** Low Intermediate High	48 (92.3%) 2 (3.8%) 2 (3.8%)	25 (96.2%) 0 1 (3.8%)	0.796
**Hasford scoring N(%)** Low High	39 (75%) 13 (25%)	20 (76.9%) 6 (23%)	0.580
**ELTS scoring N(%)** Low Intermediate High	28 (53.8%) 15 (28.8%) 9 (17.3%)	15 (57.7%) 9 (34.6%) 2 (7.7%)	0.566

#### Safety of imatinib and pioglitazone combination

Over the follow up time, edema was the most frequently reported complaint among patients in the study group (p < 0.001), whereas muscle aches were more commonly reported in the historical group (*P* = 0.026). Lower limb edema was observed in 85% (n = 22) of patients in the study group, compared to 23% (n = 11) in the historical group. Additionally, patients in the study group exhibited a significant increase in body weight over time. The mean (± SD) body weight was 78.3 ± 10.6 kg at baseline, 80 ± 11.05 kg at 3 months, and 83.6 ± 11.17 kg at 6 months, with a median weight gain of 5 kg (range: 0–10 kg). During follow-up, none of the patients in the study group exhibited abnormal elevations or reductions in random blood glucose (RBG) or fasting blood glucose (FBG) levels. Cytopenic episodes—including neutropenia, anemia, and thrombocytopenia—were observed in both groups; however, there were no significant differences in the severity grades of these hematologic abnormalities between the two groups **(**Table 2**)**.

**Table 2:** The adverse effects among both groups

	**Historical Control** ***N* = 52**	**Study group** ***N* = 26**	***p***
Allergy N (%)	0 (0%)	0 (0%)	-
Edema N (%)	12 (9.6%)	22 (76.9%)	** < 0.001**
Cytopenia N (%)	23 (44.2%)	12 (46.2%)	0.872
Muscle aches N (%)	10 (19.2%)	0 (0%)	**0.026**
GIT symptoms N (%)	5 (9.6%)	3 (11.5%)	0.256
Hepatotoxicity N (%)	11 (21.2%)	7 (26.9%)	0.569

Response and efficacy evaluation of imatinib and pioglitazone combination Q- PCR of *BCR::ABL* gene evaluation (Table 3):

**Table 3:** The BCR/ABL expression level across time among both groups

**% BCR-ABL expression level median(range)**	**Historical group**	**The Study group**	***P1***
Baseline	**88 (26–365)**	**81 (32–380)**	**0.215**
3 months	**7 (0.02–67)**	**3.65 (0.05–20)**	**0.046**
6 months	**2.0 (0.01–122)**	**0.75 (0.002–46)**	**0.049**
12 months	**0.25 (0–121)**	**0.1 (0.002–0.6)**	**0.056**
18 months	**0.06 (0–2)**	**0.02 (0–0.8)**	**0.317**
*24 months*	**0.01 (0–4.6)**	**0.02 (0–22)**	**0.586**
3 years	**0.01 (0–1)**	**0.009 (0–0.06)**	**0.410**
4 years	**0.009 (0–62)**	**0.0095 (0–0.07)**	**0.640**
5 years	**0.01 (0–37)**	**0.0095 (0–0.06)**	**0.163**

At baseline, the median *BCR::ABL* gene expression level was 81% (range = 32–380) in the study group compared to 88% (range = 26–365) in the historical cohort (***P*** = 0.215). The median *BCR::ABL* gene expression was downregulated in pioglitazone group compared to the historical group significantly at the 3rd (3.65% vs 7%; ***P*** = 0.046) and the 6th month(0.75% vs 0.2%; ***P*** = 0.049), though it was marginally insignificant at 12th month(0.25% vs0.1%: ***P*** = 0.056).However, starting from 18th month the difference between both groups was statistically insignificant (0.02% vs 0.06%; ***P*** = 0.317). This insignificant difference remained through the 24th, 36th, 48th and 60th months among both groups (Table 3).

## The achieved milestones/depth of molecular response (Table 4)

**Table 4:** The response milestones and efficacy among both groups

	**Historical N (%)** **52 (100%)**	**Study N (%)** **26(100%)**	***P***
Achieved milestones N (%)			
HR at 3 months	**41/52 (78.8%)**	**26/26 (100%)**	**0.013**
≥ CCYR at 6 months	**23/50 (46%)**	**17/26 (65.4%)**	**0.108**
≥ CCYR at 12 months	**26/35 (74.2%)**	**22/22 (100%)**	**0.009**
≥ MMR at 6 months	**9/50 (18%)**	**6/26 (23%)**	**0.598**
≥ MMR at 12 months	**19/35 (54.3%)**	**17/22 (77.3%)**	**0.080**
≥ MMR at 18 months	**19/31 (61.3%)**	**20/22 (91%)**	**0.016**
≥ MMR at 2 years	**25/29 (86.2%)**	**17/22 (77.2%)**	**0.407**
≥ MMR at 3 years	**21/27 (77.7%)**	**17/17 (100%)**	**0.036**
≥ MMR at 4 years	**19/22 (86.3%)**	**16/16 (100%)**	**0.124**
≥ MMR at 5 years	**18/19 (94.7%)**	**14/14 (100%)**	**0.383**
Depth of molecular response N (%)			
EMR 3 months	**31/52 (59.6%)**	**24/26 (92.3%)**	**0.003**
EMR 6 months	**35/50 (70%)**	**22/26 (84.6%)**	**0.163**
CMR 1y	**0/35 (0)**	**0/22 (0)**	**x**
CMR 2y	**3/29 (10.3%)**	**3/22 (13.6%)**	**0.718**
CMR 3y	**4/27 (14.8%)**	**0/17 (0)**	**0.147**
CMR 4y	**5/22 (22.7%)**	**5/16 (31.2%)**	**0.556**
CMR 5y	**5/19 (26.3%)**	**4/16 (25%)**	**0.929**
Median Time to CMR (months)	**36 (18–48)**	**48 (18–60)**	**0.670**
DMR (M4, M4.5, M5) 1 y	**6/35 (17%)**	**6/22 (27.3%)**	**0.361**
DMR (M4, M4.5, M5) 2 y	**16/29 (55%)**	**9/22 (41%)**	**0.313**
DMR (M4, M4.5, M5) 3 y	**15/27 (55.5%)**	**15/17 (88.2%)**	**0.023**
DMR (M4, M4.5, M5) 4 y	**13/22 (59%)**	**11/16 (68.7%)**	**0.542**
DMR (M4, M4.5, M5) 5 y	**13/19 (68.4%)**	**13/16 (81.2%)**	**0.387**
Median Time to DMR (Months)	**21 (6–48)**	**18 (6–36)**	**0.152**
Failing/progression to advanced stages			
Failing patients (5ys)	**27/52 (51.9%)**	**7/26(26.9%)**	**0.036**
Median(range) Time to Failure (Months)	**12(6–72)**	**6(6–24)**	**0.243**
Progressed patients (4ys)	**3/52(5.7%)**	**1/26(3.8%)**	**0.717**
Median (range) Time to Progression (Months)	**48(48–60)**	**18**	**0.157**

EMR was significantly obtained in 92.3% of the study group patients **(*P* = 0.003)**at 3 months while at 6 months the difference between both groups was not significant **(*P* = 0.163).**CCYR was achieved in 65.4% (*N* = 17), 100% (*N* = 22) of the study group patients and 46% (*N* = 23), 74.2% (*N* = 26) of the historical group patients at 6, 12 months respectively with a high significant difference only at 12 months **(*P* = 0.009)**. MMR was achieved in 23% (*N* = 6), 77.3% (*N* = 17) of the patients of the study group and 18% (*N* = 9), 54.3% (*N* = 19) of the other group at 6, 12 months respectively **(*P* = **0.598, **0.08**). However, MMR was attained significantly among patients of the study group at 18 months **(*P* = 0.016**) and 36 months **(*P* = 0.03)**. The other time points (24 months, 48 months and 60 months) did not show any further statistical significance **(*P* = 0.407, 0.124, 0.383)** respectively.

Complete molecular response (CMR) was not significantly achieved at any time point in either group, with median times to CMR of 48 months (range: 18–60) in the study group and 36 months (range: 18–48) in the control group **(*P* = 0.929).** Deep molecular response (DMR) was significantly different between the groups only at the 36-month time point **(*P* = 0.023),** while no significant differences were observed at other time points. The median time to DMR was 18 months (range: 6–36) in the study group and 21 months (range: 6–48) in the control group **(*P* = 0.152).**

## Treatment failure and transformation to advanced stages:

During the study period, first-line treatment failure occurred in 7 patients (26.9%) in the study group compared to 27 patients (51.9%) in the control group, with a statistically significant difference **(*P* = 0.036).** The median time to treatment failure was 6 months (range: 6–24) in the study group and 12 months (range: 6–72) in the historical group **(*P* = 0.243).** Regarding disease progression, only one patient (3.8%) in the study group progressed to the accelerated phase (AP) after 18 months of therapy, compared to three patients (5.8%) in the control group, with no statistically significant difference **(*P* = 0.717).**

## Detection of the *CITED2and HIF2*α genes expression levels (pre-treatment, post-treatment in the study group and in the healthy control group) (Tables 5,6)

**Table 5:** Comparison between CITED2, HIF2a gene expression level between healthy group and study group patients

Gene expression		Healthy Control (*N* = 12)	Study *N* = 25		*p1*	*p2*	*p3*
			**Pre treatment**	**Post treatment**			
**CITED 2**	**Median**	**0.96**	**276.3**	**2.6**	** < 0.001**	**0.005**	**0.119**
	**range**	**0.082–2.2**	**1–241,221** **.7**	**0.006–86475.3**			
**HIF2**	**Median**	**0.38**	**2.7**	**1**	**0.004**	**0.026**	**0.082**
	**range**	**0.03–4.68**	**0.041–1255647.4**	**0.006–47.2**			

***P1***: comparing the gene expression between the healthy control and the study group patients pretreatment,

***P2***: comparing the gene expression pre and post treatment within the study group patients

***P3***: comparing the gene expression between the healthy control and the study group patients posttreatment

**Table 6:** Comparison the CITED2, HIF2a post- treatment gene expression level among responders and non-responders

Gene expression		Study *N* = 25			*P*
		Responders *N* = 21		Non responders *N* = 4	
	Pre-treatment expression	Median	261.4	3145	0.630
CITED 2		Range	1–241,221.7	1–14,972.2	
	Post-treatment	Median	2.2	92.6	0.235
	expression	Range	0.01–103.25	0.01–86475.27	
		Median	1.6	19.7	
	Pre-treatment expression	Range	0.041–32768	1–1,255,647.4	0.373
HIF2α	Post-treatment	Median	1	0.8	0.23
	expression	Range	0.01–47.18	0.02–1	2

**Fig. 1:**
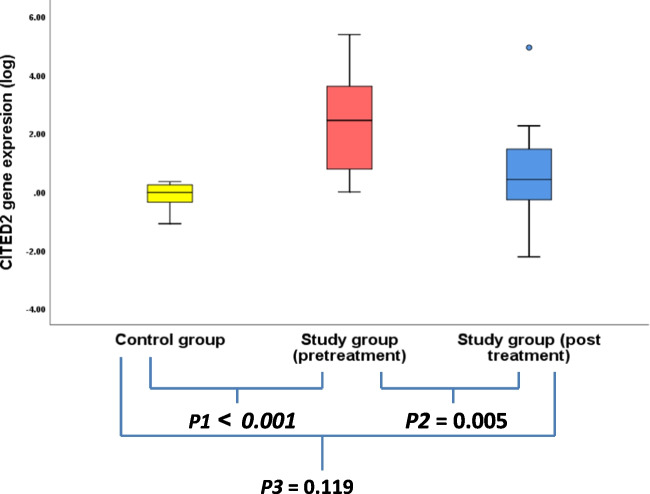
CITED2 gene expression among healthy control and within study group

CITED2 gene expression was measured in study group patients prior to treatment, revealing a median expression level of 276.3 (range: 1–241,221.7). This baseline level was significantly elevated—approximately 288-fold—compared to age- and gender-matched healthy controls (***P₁*** < 0.001). Following the combined treatment regimen, *CITED2* expression decreased significantly to a median of 2.6 (range: 0.006–86,475.3) (***P₂*** = 0.005), with no statistically significant difference compared to the control group (***P₃*** = 0.119) (Fig. 1).

**Fig. 2:**
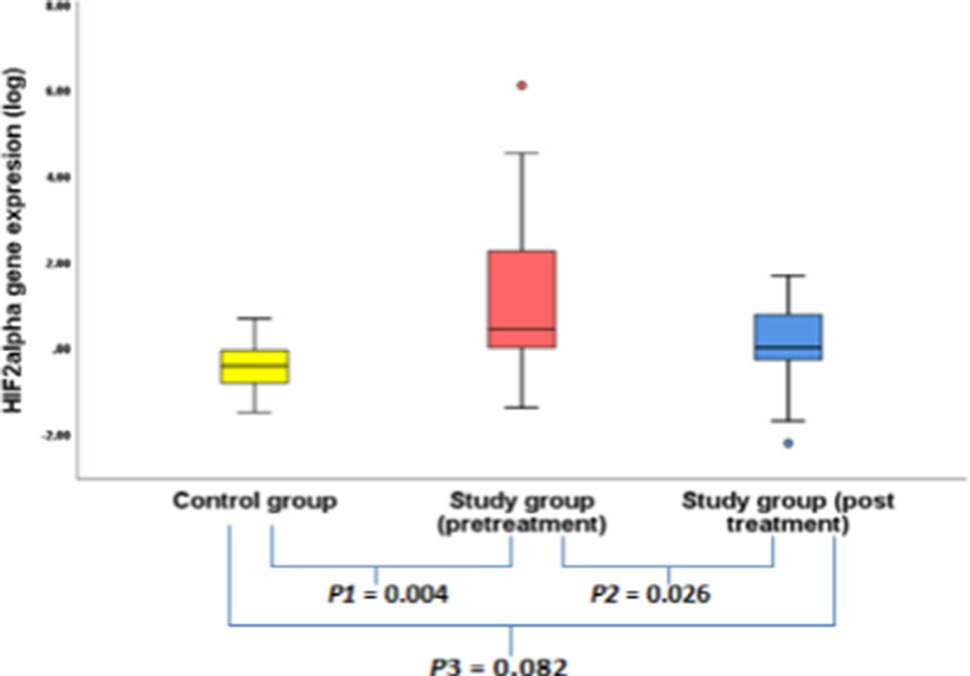
HIF2α expression among study group and healthy control

*HIF2α* gene expression was assessed in patients before treatment revealing a median of 2.7 (range = 0.041–1255647). This pretreatment expression level is significantly higher (7 folds) when compared to the healthy control group **(*P1* = 0.004)**. The gene expression level also decreased significantly after receiving the combined treatment to a median of 1 (range = 0.006–47.2) **(*P2* = 0.026)** that was not significantly different from the normal subject’s level **(*P3* = 0.082) (**Fig. 2**)**. No significant associations were found between the median pretreatment genes expression level with demographic, and laboratory data among study groups. Both genes showed no significant difference between the pre-treatment and the post-treatment levels among responders and non-responders.

## Discussion

Although the quantum leap in the response spectrum after using TKIs in treating CML, TKIs alone (even the new generations) are still unable to completely eliminate the LSCs and cure CML [14, 15]. LSCs act as the game player in TKIs resistance and disease relapse [3, 4]. Combining pioglitazone (FDA approved PPARγ agonist in treating T2DM) with imatinib was reported to push LSCs out of their quiescence that can deplete the CML stem cell pool [5]. To our knowledge, there are no population-based studies of this combination therapy in newly diagnosed CML patients. We aimed -in the study- to evaluate the safety and efficacy of this combination as upfront treatment line whether it could help to obtain deeper and may be faster molecular responses or not.

In the present study, 26 newly diagnosed chronic phase Philadelphia chromosome-positive (Ph + CP) chronic myeloid leukemia (CML) patients—without advanced disease or organ dysfunction—were treated with a combination of imatinib (400 mg) and pioglitazone (15 mg) daily for six months. Unlike previous studies that evaluated pioglitazone at higher doses (30–45 mg daily) and longer durations [5, 10, 16], our trial adopted a shorter six-month course at a lower dose in order to minimize potential adverse effects while assessing its therapeutic efficacy in the upfront therapy setting [5].

Regarding the safety profile of the combination therapy, pioglitazone was generally well tolerated among the majority of patients. No episodes of hypoglycemia or grade 3–4 adverse events were observed. The most commonly reported adverse effects were edema and increased body weight. Edema of varying severity was observed in 84.6% of patients, predominantly presenting as grade 2 pedal edema. This was effectively managed with furosemide (40 mg), and no patient discontinued treatment due to adverse effects. In comparison, a clinical trial conducted at Emory University (NCT02730195) reported pedal edema in 55% and facial edema in 11.1% of participants. In the ACTIM trial, one patient withdrew due to grade 2 edema [10], while another study reported discontinuation of pioglitazone in two patients due to pedal edema [16]. The EDI-PIO trial noted a need to reduce the pioglitazone dose to 30 mg on alternate days in response to worsening periorbital and limb edema [17].

With respect to weight gain, a median increase of 5 kg (range: 1–10 kg) was observed in 88.4% of patients. Due to the absence of body weight data in the control group's retrospective records, direct comparison was not feasible. In the EDIPIO trial, a statistically significant increase in mean body weight was reported following pioglitazone treatment (83.7 ± 22 kg; *P* = 0.003), likely attributable to the higher doses used (30–45 mg daily) [17]. In contrast, the French study reported a non-significant weight increase in 50% of patients (*N* = 12) [10], while Goyal et al. observed no weight gain in their cohort [16]. These discrepancies may be influenced by ethnic and dietary differences across study populations.

Anemia and neutropenia were observed in 57.6% and 53.8% of patients, respectively, while thrombocytopenia was reported in 38.4%. These hematologic toxicities were managed effectively by temporarily withholding treatment for up to 1–2 weeks [13]. In comparison, Rousselot et al. reported a statistically significant incidence of anemia (*P* = 0.03) after 12 months of therapy [10], whereas the Emory University trial (NCT02730195) documented only a single case of anemia over a 6-month period. Goyal et al. did not observe any reduction in hemoglobin levels in their study population [16].

In terms of efficacy, a significant reduction in *BCR::ABL1* transcript levels was observed in both groups at 3 and 6 months. Despite comparable clinicopathological characteristics and risk profiles between the groups, the study cohort demonstrated a 12-month complete cytogenetic response (CCyR) rate of 100% (N = 22), which was significantly higher than that of the imatinib-only group (*P* = 0.009). Additionally, a greater proportion of patients in the study group achieved a 12-month major molecular response (MMR) (65.3%) compared to 50% in the historical control group, although this difference did not reach statistical significance [Table 4].

At 6 months, 23% of patients in the combination therapy group achieved MMR, a rate that was not statistically significant compared to the control group (*P* = 0.598). This finding is consistent with Goyal et al., who reported a 22.5% MMR rate when pioglitazone was used as an adjunctive therapy [16]. Notably, previous in vivo studies assessing the efficacy of the imatinib–pioglitazone combination were conducted in CML patients who had already received treatment but had not yet achieved MMR or complete molecular response (CMR), aiming to deepen their molecular response [5, 10, 16, 18]. In contrast, our study employed pioglitazone as part of the initial, upfront treatment strategy.

To evaluate the potential benefit of using the combination as upfront therapy, we compared our findings with outcomes from various imatinib trials. Studies using standard-dose imatinib (400 mg/day) have reported 12-month CCyR and MMR rates ranging from 55–66% and 22–38%, respectively, while high-dose imatinib (600–800 mg/day) achieved rates of approximately 65% for CCyR and 45–55% for MMR. Additionally, the three largest trials employing second-generation TKIs as first-line treatment—ENESTnd, DASISION, and BEFORE—reported 12-month CCyR rates of 80%, 77%, and 77%, and corresponding MMR rates of 44%, 46%, and 47%, respectively [19, 20]. Notably, the 12-month MMR rate observed in our study is also comparable to that reported in the EPIC trial [21].

A detailed analysis of molecular responses demonstrated that a significantly greater proportion of patients treated with the combination therapy achieved early molecular response (EMR) at 3 months compared to the imatinib-only group (92.3% vs. 59.6%, *P* = 0.003). These results are comparable to EMR rates reported in major frontline TKI trials—ENESTnd (91%), DASISION (84%), and BEFORE (75%) [22]. Moreover, although not statistically significant, 9% of patients in the combination group achieved MR^4.5 at 12 months. This rate aligns with previously reported 12-month MR^4.5 rates of 9% for high-dose imatinib, 11% for nilotinib, 5% for dasatinib, and 8% for bosutinib [19]. While these trials involved larger patient cohorts, achieving similar molecular outcomes with fewer adverse effects and lower treatment costs highlights the potential of this combination as a viable therapeutic option, particularly in resource-limited settings [23].

These promising early results encouraged us to monitor long-term outcomes with the aim of identifying a potentially accelerated path to treatment-free remission (TFR). However, the findings did not meet these expectations. Beyond the first year, the decline in *BCR::ABL1* transcript levels was not statistically significant in either cohort [Table 3]. Similarly, the proportions of patients achieving complete molecular response (CMR) and deep molecular response (DMR) over time were largely comparable between the groups, with the exception of a statistically significant difference in the 3-year DMR rate (*P* = 0.023) [Table 4].

The 5-yearfollow up results were not different among the two groups as well. The M4.5 rate was 37.5% that is less than 42% and 54% reported results from DASISION andENESTnd [19].These responses may not persist due to the short duration of pioglitazone treatment or the small sample size (after excluding non-responders). The potential efficacy may be enhanced with higher doses, extended duration or by implying pulsed (on/off) therapeutic cycles. Considering an alternative perspective 'Treatment Failure,' the study group demonstrated significantly fewer failing (*P* = 0.03) and non-significant less progressed patients. This may suggest a potential hidden benefit of the combination therapy that possibly improving long-term disease control and eligibility for treatment-free remission planning.

The early results were supporting the suggested proposal about the synergism between the PPARγ agonists and TKIs in affecting the CML LSCs through inhibition of STAT5 and its target genes [8, 10, 24]. Therefore, we tested the pretreatment and post treatment gene expression levels of *CITED2* and *HIF2α.* Both genes were found to be significantly overexpressed in the pretreatment samples. The over expression has been already detected in many pretreated CML and AML patients for CITED2 gene [25–27] and for the HIF2α in myeloid leukemia cell lines U937, HL60 and THP1 [28]. These studies emphasize their role in regulating the oncogene-induced quiescent LSCs pool implying a worse prognosis [29–32].

Notably, the expression levels of both genes significantly decreased in post-treatment samples (*P* = 0.005 and *P* = 0.026, respectively), and were not significantly different from those observed in the healthy control group (*P* = 0.11 and *P* = 0.08, respectively). These findings suggest a potential effect of the combination therapy on leukemic stem cells (LSCs). This observation aligns with the results reported by Prost et al. [5]. In contrast, Lopès et al. found no significant changes in *HIF2α* and *CITED2* expression before and after pioglitazone treatment at 3 and 6 months following imatinib discontinuation [33]. In our study as well as Lopès et al. both genes expression was assessed in PBMNCs [5], however Prost et al. was testing their expression in bone marrow–derived CD34⁺ progenitors [33]. Such comparisons across studies using different cellular populations (e.g., PBMCs vs CD34⁺ progenitors) should be interpreted with caution.

Further analysis revealed no significant associations between pretreatment median levels of *CITED2* and *HIF2α* gene expression and the demographic, clinical, or laboratory parameters within the study group. These findings are consistent with previous reports showing a lack of correlation between the expression of these genes and clinical or laboratory characteristics in both myeloid leukemia and various solid tumors [30, 34, 35]. Additionally, no significant differences were observed in post-treatment gene expression levels between responders and non-responders. This may suggest a limited prognostic value of these genes and highlights the need for further investigation in larger patient cohorts [36].

## Limitations

This study has several limitations that should be acknowledged. First, the small sample size (*n* = 26) limits the statistical power and generalizability of the findings. Additionally, the use of a historical control group rather than a randomized, contemporaneous control introduces potential selection and information biases, and the lack of randomization and blinding increases the risk of bias in outcome assessment. Moreover, although short-term responses appeared promising, the long-term outcomes did not align, indicating a lack of durability in response. The wide variability observed in *CITED2*and *HIF2a* gene expression levels may reflect inter-patient differences or inconsistencies in measurement techniques, and the clinical relevance of the gene expression changes remains unclear, as the study did not directly assess leukemic stem cell burden. Finally, the study's focus on newly diagnosed chronic phase patients limits the applicability of results to broader CML populations.

## Summery and conclusion

In our study, adding pioglitazone to imatinib in newly diagnosed CML was associated with faster molecular responses compared to historical controls. By 3rd and 6th months, patients in the combination group demonstrated significantly lower median BCR::ABL1 transcript levels, and higher rates of EMR. Achieving MMR at 18 months and at three years was also more frequent in the study group. The regimen was generally well tolerated, with no unexpected toxicities observed at the low dose of pioglitazone (15 mg daily). This short-term molecular benefit unfortunately lacked long-term durability as the rates of CMR and DMR were comparable between groups at later time points along 60 months follow-up though showing a lower incidence of treatment failure. These responses may not persist due to the short duration of pioglitazone treatment or the small sample size. The potential efficacy may be enhanced with higher doses, extended duration or by implying pulsed (on/off) therapeutic cycles. Although *CITED2* and *HIF2α* expression decreased with treatment, these changes did not align with BCR::ABL1 transcript levels, suggesting limited utility as molecular markers. Their potential prognostic value, however, warrants further investigation in larger studies.

## Data Availability

The clinical and genetic data supporting the findings of this study are not publicly available due to institutional restrictions. However, the data do not contain identifiable human subject information, and there are no legal or ethical limitations preventing their use. Data may be made available to qualified researchers upon reasonable request and subject to approval by the corresponding institutional review board and data access committee. Requests for access to the data can be submitted through the *Annals of Hematology* journal in accordance with its data access policies.
